# Crystal and Electronic Structure of Oxygen Vacancy Stabilized Rhombohedral Hafnium Oxide

**DOI:** 10.1021/acsaelm.2c01255

**Published:** 2023-01-26

**Authors:** Nico Kaiser, Young-Joon Song, Tobias Vogel, Eszter Piros, Taewook Kim, Philipp Schreyer, Stefan Petzold, Roser Valentí, Lambert Alff

**Affiliations:** † Advanced Thin Film Technology Division, Institute of Materials Science, TU Darmstadt, Alarich-Weiss-Str. 2, 64287Darmstadt, Germany; ‡ Institute for Theoretical Physics, Goethe-University Frankfurt, Max-von-Laue-Straße 1, 60438Frankfurt am Main, Germany

**Keywords:** *r*-HfO_1.5_, hafnium oxide, cubic, rhombohedral, polar phase, oxygen vacancies, density of states, bandgap

## Abstract

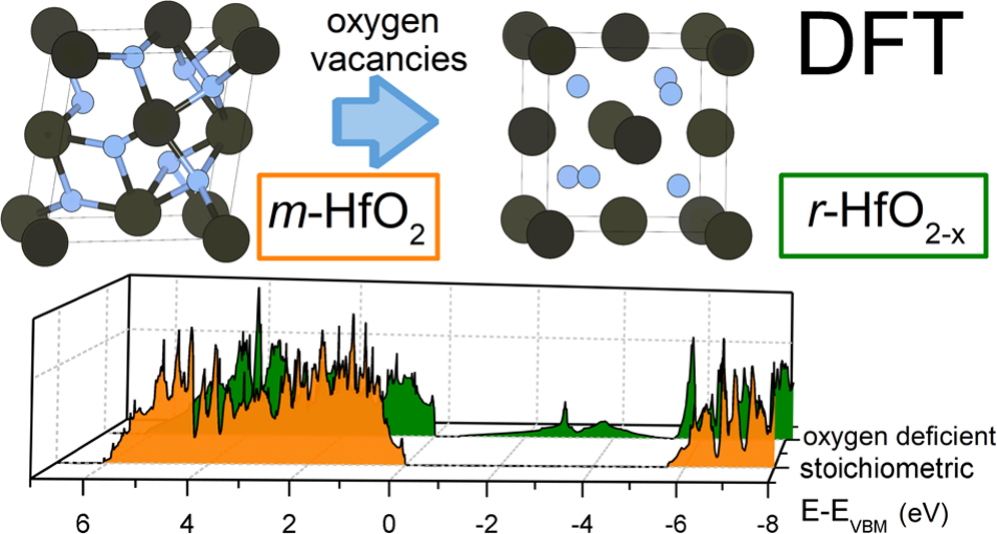

Hafnium oxide is an outstanding candidate for next-generation nonvolatile memory solutions such as OxRAM (oxide-based resistive memory) and FeRAM (ferroelectric random access memory). A key parameter for OxRAM is the controlled oxygen deficiency in HfO_2-*x*_ which eventually is associated with structural changes. Here, we expand the view on the recently identified (semi-)conducting low-temperature pseudocubic phase of reduced hafnium oxide by further X-ray diffraction analysis and density functional theory (DFT) simulation and reveal its rhombohedral nature. By performing total energy and electronic structure calculations, we investigate phase stability and band structure modifications in the presence of oxygen vacancies. With increasing oxygen vacancy concentration, the material transforms from the well-known monoclinic structure to a (pseudocubic) polar rhombohedral *r*-HfO_2–*x*_ structure. The DFT analysis shows that *r*-HfO_2–*x*_ is not merely epitaxy-induced but may exist as a relaxed compound. Furthermore, the electronic structure of *r*-HfO_2–*x*_ as determined by X-ray photoelectron spectroscopy and UV/Vis spectroscopy corresponds very well with the DFT-based prediction of a conducting defect band. The existence of a substoichiometric (semi-)conducting phase of HfO_2–*x*_ is obviously an important ingredient to understand the mechanism of resistive switching in hafnium-oxide-based OxRAM.

## 1 Introduction

Hafnium oxide is relevant for both OxRAM and FeRAM, and therefore directly connected to emerging fields in data processing such as in-memory computing or Internet of Things.^1−4^ Further, as multibit gradual switching can be observed in a multitude of RRAM device configurations, it appears directly applicable for neuromorphic (“brain-like”) computing where single devices mimic the behavior of synapses.^2,5,6^ Hafnium oxide was previously investigated and optimized as a high-*k* solution for semiconductor manufacturing. Therefore, its complementary metal oxide semiconductor (CMOS) compatibility is already well established.^7−9^

One key parameter that crucially affects the functionality of such oxide-based devices is oxygen deficiency. While very low levels of oxygen vacancies mostly affect the leakage performance of the dielectric, higher levels of deficiency are known to govern the resistive switching behavior. This regime is either accessed by the use of scavenging layers, which deprive a stoichiometric layer from oxygen after heating,^10^ or by direct control of stoichiometry using oxygen engineering in a physical vapor deposition process. In this region, the switching characteristics can be tuned to achieve favorable behavior like low forming or gradual multibit switching.^5,6^ (Local) High levels of oxygen deficiency lead to conducting hafnium oxide,^11−15^ which plays a key role in the switching mechanism where a conductive filament is formed and ruptured by reversible redox processes.^16,17^

For TiO_2_-based OxRAM, a conducting substoichiometric Magnéli phase (Ti_4_O_7_) has been observed by transmission electron microscopy (TEM).^18^ This finding suggests that a conducting filament in transition-metal oxides can be formed by substoichiometric phases with a defined crystal structure.

In the context of hafnium oxide, electrically conductive sub-oxides like Hf_2_O_3_, Hf_6_O have been suggested by materials modeling.^11,12,14,15,19^ However, these substoichiometric phases have not been observed in memristive devices. Here, we go the reversed way by determining the exact structure of the experimentally observed oxygen-deficient phase of HfO_2–*x*_ and confirm it in a joint experimental and theoretical approach.

For this purpose, we present a density functional theory (DFT) perspective on the recently identified substoichiometric phase, namely, the low-temperature pseudocubic phase of hafnium oxide (LTP *c*-HfO_2–*x*_).^20^ While already in the previous work a slight rhombohedral distortion from the cubic structure was discussed, we now pinpoint the exact rhombohedral nature of the phase (*r*-HfO_2–*x*_) by additional X-ray diffraction (XRD) analysis and DFT calculations. Previously, this phase was analyzed by a wide range of methods, including XRD, X-ray photoelectron spectroscopy (XPS), UV–vis transmission spectroscopy, and electrical measurements, and it was shown to be stabilized from the stoichiometric monoclinic phase via oxygen vacancies.^20^ Further, this transformation was found to be accompanied by an emerging defect band and the development of significant electrical conduction. In the present work, we investigate this phase with density functional theory-based calculations invoking hybrid exchange–correlation functionals and confirm the experimental results of the oxygen content-dependent transformation of the crystal and band structure. The discussed results are therefore of particular significance for understanding the defect chemistry and electronic structure of substoichiometric hafnium oxide.

## 2 Methods: Experiments and Calculations

We performed DFT-based *ab initio* calculations on both monoclinic and cubic/rhombohedral phases of HfO_2–*x*_ (*x* = 0, 0.25, 0.5) using the projector augmented wave method^21^ as implemented in the Vienna Ab initio Simulation Package (VASP).^22^ The revised generalized gradient approximation (GGA)^23,24^ was adopted as the exchange–correlation functional. Additionally, for a better description of the gaps in the electronic structure, we also employed the Heyd–Scuseria–Ernzerhof hybrid functional (HSE06)^25^ as implemented in VASP. The mixing parameter α of the Hartree–Fock exchange functional and the screening parameter μ for HSE06 were chosen to be 0.25 and 0.2, respectively. The cutoff energy for the plane wave basis set was set to be 500 eV. The Brillouin zone was sampled by a 10 × 10 × 10 *k*-mesh for structural relaxations as well as HSE06 calculations. A 12 × 12 × 12 *k*-mesh for the standard GGA calculations was considered. All crystal structures of HfO_2–*x*_ were fully relaxed within GGA until the Hellmann–Feynman forces were smaller than 1 meV/Å.

All thin films were grown on *c*-cut Sapphire substrates utilizing a custom-designed molecular beam epitaxy system with a base pressure of 10^–9^ mbar. The elemental sources for deposition consisted of quartz crystal microbalance (QCM) controlled electron beam evaporated hafnium and mass-flow controlled oxygen, which was stimulated into a plasma by a constant radio frequency power of 200 W. For all depositions, a constant thermocouple-calibrated substrate temperature of 320 °C was set. By calibration via X-ray reflectometry (XRR), a consistent film thickness of 20 ± 2 nm was achieved throughout the series. XRD and XRR measurements were conducted with a Rigaku SmartLab diffractometer in parallel beam configuration, equipped with a Ge(220) double-bounce monochromator. Both XRD and XRR measurements were performed employing Cu K_α_ radiation. For pole-figure measurements, the Ge(220) monochromator was changed to a graphite monochromator, to improve the signal-to-noise ratio. All XPS measurements have been conducted *in vacuo* as the deposited thin films have been transferred via a vacuum suitcase equipped with an ion getter pump and ion gauge to guarantee a transfer in uninterrupted ultrahigh-vacuum conditions. The XPS spectra have been obtained via a PHI VersaProbe 5000 spectrometer (Physical Electronics), where the photoelectrons were excited with monochromatic Al K_α_ radiation and collected at an escape angle of 75°. To avoid charging effects on the insulating Sapphire substrates, the spectra were recorded utilizing dual-beam charge neutralization. The experimental Fermi level position was estimated by a gold-calibrated reference measurement from a sample that was grown in the same deposition process but on a conducting Si/TiN substrate (instead of insulating Sapphire). Optical spectroscopy measurements have been conducted with a Cary 7000 UV–vis system from Agilent. These UV–vis measurements were performed in transmission mode in single-beam configuration. The corresponding transmission spectra of the substrates have been measured separately and deducted from the total spectra.

## 3 Results and Discussion

### 3.1 Oxygen Vacancy-Induced Phase Transition from Monoclinic to Rhombohedral Hafnium Oxide

**Figure 1:**
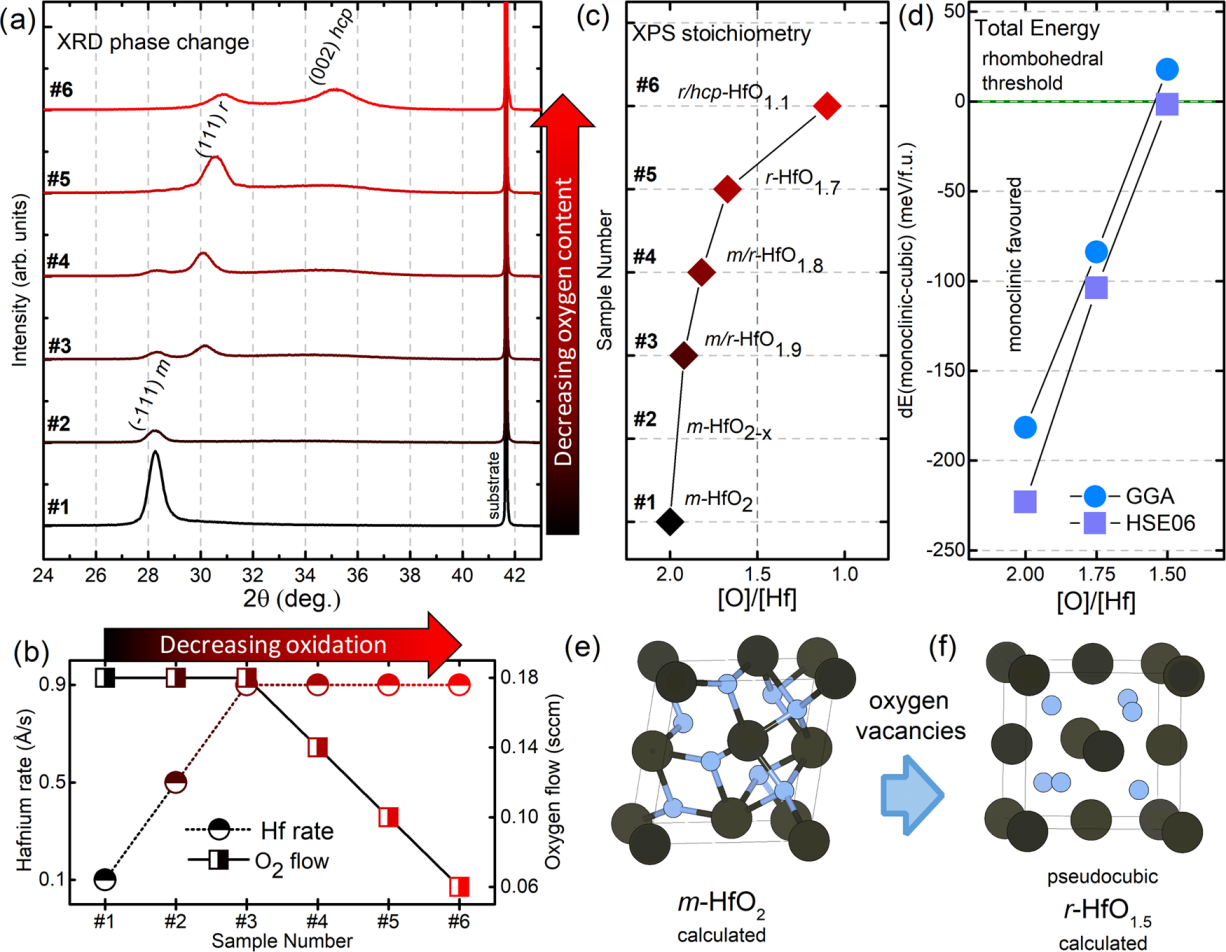
(a) Oxygen content-dependent structural transformation from monoclinic *m*-HfO_2_ (sample #1) to rhombohedral hafnium oxide (*r*-HfO_1.7_) (sample #5). For sample #6, the intensity of the reflection corresponding to *r*-HfO_1.7_ decreases and a further transformation to *hcp*-HfO_2–*x*_ emerges. (b) Corresponding MBE deposition parameters for each sample with consistent decreasing oxidation conditions from sample #1 to #6. (c) Stoichiometry as determined by XPS measurements indicating a composition close to [O]/[Hf]= 1.7 (#5) for *r*-HfO_2–*x*._ (d) Comparison of the total energy difference between monoclinic HfO_2–*x*_ and rhombohedral HfO_2–*x*_ from [O]/[Hf] = 2.0 to 1.5, confirming an oxygen content-dependent trend and critical composition for a structural transformation to a rhombohedral phase at [O]/[Hf] = 1.5, close to the XPS measurement. (e, f) Sketches of the calculated structures corresponding to *m*-HfO_2_ and *r-*HfO_1.5_.

To investigate the influence of oxygen deficiency on the crystal structure of hafnium oxide, six thin films have been grown at a CMOS-compatible temperature of 320 °C under decreasing oxidation conditions. All samples have been grown onto *c*-cut Sapphire to allow epitaxial growth and to provide an insulating, wide-bandgap material for UV–vis transmission measurements. The structural transformations are shown in Figure 1a by XRD. The 2θ/ω out-of-plane measurements show for highest oxidation conditions (black) that only the monoclinic phase of stoichiometric HfO_2_ is present.^26^ By reducing the oxidation conditions (toward red), the monoclinic phase vanishes, while a second phase appears, which we identified as a rhombohedral phase of hafnium oxide as will be discussed in detail in Section 3.2. The reflection in oxygen-deficient samples is often attributed to a tetragonal or orthorhombic phase by referring to the high-temperature or high-pressure phases of HfO_2_.^27−29^ Recently, the tetragonal and orthorhombic structures were excluded by a detailed investigation, and a pseudocubic phase (designated LTP *c*-HfO_2–*x*_) was identified.^20^ Here, we show that the apparent pseudocubic phase relaxes to a rhombohedral phase *r*-HfO_2–*x*_, which becomes most prominent for the deposition conditions of sample #5, with no contributions from the monoclinic structure. For sample #6, a further transition to a structure close to the metallic hexagonal structure is observed. Previously, it was shown by combined XRD and XPS measurements that this structure has a modified hexagonal lattice with oxygen interstitials *hcp*-HfO_2–*x*_.^20^ This observation is in good agreement with different DFT publications where a metallic hexagonal phases with oxygen interstitials at octahedral positions are found to be the most likely modifications as indicated by total energy calculations.^11,14,15^ XPS estimations suggest that this hexagonal phase can host oxygen up to at least [O]/[Hf] = 0.7, which is in good agreement with the maximum capacity for octahedral occupation at [O]/[Hf] = 1.0.^20^Figure 1b shows the corresponding deposition conditions of all six samples with the rate of evaporated metallic hafnium and the gas flow through the oxygen plasma source.

Figure 1c shows the elemental composition of the whole sample series as determined by XPS from area matching of the Hf 4*f* and O 2*p* emission lines.^20^ While the measurement conditions are listed in the Section 2, the corresponding spectra with additional fitting information are listed in the Supporting Information (see Figure S3). A more comprehensive discussion on the corresponding spectra is given elsewhere.^20^ Starting with [O]/[Hf] = 2 for stoichiometric monoclinic hafnium oxide in sample #1, a continuous decrease of oxygen content up to [O]/[Hf] = 1.1 for sample #6 is observed. For *r*-HfO_2–*x*_ containing thin films, the XPS-estimated overall oxygen content ranges from [O]/[Hf] = 1.9 (#3) to 1.1 (#6) with specifically 1.7 for the most phase-pure sample of *r*-HfO_2–*x*_ (#5).

Note that the linear change of growth parameters as displayed in Figure 1b does not translate to a linear trend in the samples oxygen content. An explanation for this behavior is given by the complex interrelation of main parameters with secondary parameters in a molecular beam epitaxy system. Any change of the gas flow gives rise to a change in the plasma intensity (at constant forward power), which also does not follow a linear behavior. Similarly, the interaction of the plasma with the evaporated hafnium results in a specific oxygen partial pressure in the system, which then also does not follow a proportional dependence with the gas flow (compare Figure S1 in the Supporting information).

To compare these experimental results on the oxygen-dependent structural transformation with DFT simulations, we performed total energy calculations on fully relaxed crystal structures with oxygen vacancies. Therefore, we first performed structure relaxations on stoichiometric monoclinic and cubic HfO_2_ in the space group *P*2_1_/*c* and *Fm*3̅*m*, respectively, within GGA. Based on the fact that there are two formula units in the unit cell of HfO_2_, HfO_1.75_ and HfO_1.5_ can be achieved by taking one and two oxygen atoms out of the unit cell. For HfO_1.75_, there are eight possibilities to choose one out of eight oxygen atoms. However, due to the symmetry of cubic and monoclinic HfO_2_, they were reduced to one candidate for *c*-HfO_1.75_ and two candidates for *m*-HfO_1.75_. On the other hand, for HfO_1.5_, 3 and 10 candidates exist out of 28 possibilities for cubic and monoclinic HfO_2_, respectively. For the structure relaxations, volume, atomic positions, and shape of the cell were fully relaxed.

Interestingly, the oxygen vacancies in *c*-HfO_2_ induce a tiny rhombohedral distortion after the structure optimization. As a result, the space group of *c*-HfO_1.75_ and *c*-HfO_1.5_ turns into **R**3**m** (a rhombohedral space group); on the other hand, that of *m*-HfO_1.75_ and *m*-HfO_1.5_ goes to the triclinic system by lowering symmetries. Since the variations from the monoclinic to triclinic structures are small, we remain in the following with the designation ″monoclinic″ also for the triclinic distorted unit cells to avoid confusion (the exact structural parameters of all calculated phases are listed in the Supporting Information). The calculated structures for both *m*-HfO_2_ and *r*-HfO_1.5_ are shown in Figure 1e,f, respectively, while the structural data of lattice parameters and atomic positions are listed in Table S1 in the Supporting Information. A direct structural comparison between experimental results and theoretical calculations on the rhombohedral phase is provided in Section 3.2.

Figure 1d shows a comparison between the total energy calculations of monoclinic HfO_2–*x*_ and (pseudo)cubic HfO_2–*x*_ with changing oxygen content for both structures from [O]/[Hf] = 2 to 1.5, confirming the oxygen dependency of the monoclinic-to-rhombohedral phase transformation. While clearly for [O]/[Hf] = 2 the monoclinic structure is favored, for both GGA and HSE06 simulations, the total energy difference either cuts or is very close to the threshold to the rhombohedral transformation at [O]/[Hf] = 1.5. This observation is in good agreement with the previously discussed experimental results where a ratio close to [O]/[Hf] = 1.7 was estimated. Similar calculations have been performed by McKenna et al. who also performed total energy calculations on oxygen-dependent phase formations in hafnium oxide but found that up to a reduction to [O]/[Hf] = 1.3, the monoclinic phase was favored over a tetragonal phase.^11^ From the overall calculations, it was concluded that in oxygen-deficient hafnium oxide, a phase separation between monoclinic and hexagonal hafnium oxide would be most favorable.^11^ However, as already discussed in this section, our results show from an experimental and theoretical perspective that the formation of a rhombohedral structure (*r*-HfO_2–*x*_) as an intermediate phase is evident. Additionally, this phase has been remeasured via XRD after 3 years, showing no indication of degradation, therefore confirming its significant stability.

### 3.2 Structural Comparison between Stoichiometric Cubic (*c*-HfO_2_) and Deficient Rhombohedral Hafnium Oxide (*r*-HfO_2–*x*_)

The structural identification of oxygen-deficient hafnium oxide is not a trivial task as such compounds are exclusively synthesized in form of thin films where XRD analysis is generally prone to peak broadening and texture variation. This results in the inapplicability of reliable crystal identification approaches like the Rietveld method. Hafnium oxide structures like the high-temperature tetragonal (**P**4_2_/**nmc**) and cubic (*Fm*3̅*m*) phases or the orthorhombic high-pressure phase (**Pbca**)^26,30,31^ are highly interrelated, resulting in significant uncertainties in peak indexing and phase classification. Therefore, oxygen-deficient hafnium oxide is often inconsistently indexed by bulk compounds of stoichiometric hafnium oxide polymorphs.^27−29^ However, using highly epitaxial growth conditions in combination with comprehensive XRD analysis, we identified a cubic phase with a slight rhombohedral distortion as an intermediate phase in the transition from stoichiometric monoclinic hafnium oxide to metallic hafnium with oxygen interstitials. To draw a distinction to the high-temperature cubic phase (**Fm**3̅**m**), which is induced upon heating of stoichiometric hafnia, the oxygen vacancy-induced phase was named low-temperature phase of cubic hafnium oxide (LTP *c*-HfO_2–*x*_).^20^ The precise XRD analysis was possible by evaluation of potential peak splitting around the (200) reflex by measuring multiple 2θ scans at defined ψ and φ angles. Hereby, peak splitting (indicative of a tetragonal or orthorhombic phase) could be unambiguously excluded and the (200) reflex was confirmed as a single lattice plane. However, already in this previous publication, a slight rhombohedral distortion was concluded from the lattice spacing’s of the (111) and (200) planes.^20^ Here, we pinpoint the exact rhombohedral nature of this pseudocubic phase by additional XRD analysis and calculations.

**Figure 2:**
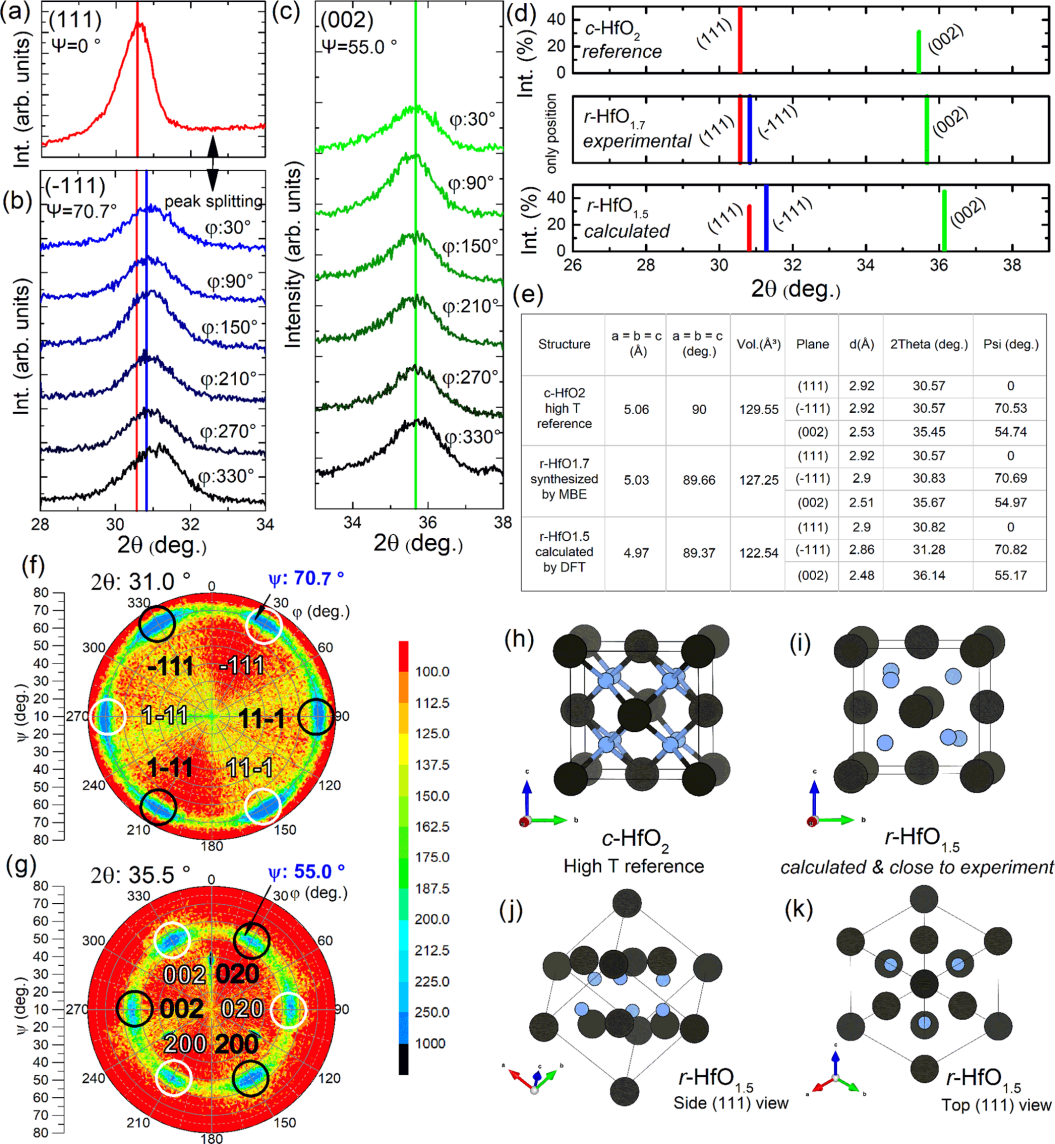
(a) Measured (111) out-of-plane lattice constant. (b) Peak shift of ∼0.25° in 2θ for the (1̅11) lattice planes, confirming the rhombohedral distortion of the investigated phase. (c) Corresponding (002) lattice parameters. All vertical lines from (a–c) indicate the corresponding peak maxima and satisfy the mathematical expression for a rhombohedral system with α = β = γ = 89.66° (close to cubic). (d) Hypothetical powder diffraction patterns of the stoichiometric cubic (high-temperature) phase *c*-HfO_2_, rhombohedral *r*-HfO_1.7_ from measurement and *r*-HfO_1.5_ obtained from the calculations. Note that the deficient phases show an oxygen vacancy-induced rhombohedral transformation from the cubic reference for both measurement and simulation as indicated by the (111), (1̅11) peak splitting. (e) Corresponding table with detailed information about lattice parameters and relevant lattice planes. (f, g) pole figures showing highly epitaxial growth with two rhombohedral domains being offset by 60°. (h) Reference structure of the high-temperature phase of cubic hafnium oxide (**Fm*3̅*m**) (i) simulated *r*-HfO_1.5_ with (j) (111) growth direction from the top and (k) from the side view (compare Table S1).

Figure 2a shows the measured reflection of the out-of-plane (111) lattice parameter. Figure 2b shows the (1̅11) reflections (blue vertical line as reference), with a slight shift of ∼0.25° in 2θ compared to the (111) reflection (red vertical line as reference). This peak splitting between (111) and (1̅11) is a smoking gun evidence of the rhombohedral nature of the phase. Finally, Figure 2c shows the corresponding (002) lattice planes. The vertical lines in all graphs highlight the peak maxima. All (1̅11) and (002) planes are expected to show the same peak maxima, respectively. Note that the single (1̅11) reflection at φ: 330° shows a small offset by ∼0.2° in 2θ, which is most likely a consequence of a different contribution, overlaying the signal from this (1̅11) plane (further details are listed in the Supporting Information).

The positions of all three lines (red, blue, and green) satisfy the mathematical relation for a rhombohedral cell^32^
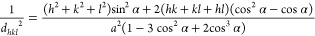
with α = β = γ = 89.66°, confirming the rhombohedral nature of the phase.

Figure 2d shows the powder diffraction patterns for stoichiometric cubic hafnium oxide (PDF 04-011-9018) for reference, as well as patterns for the oxygen-deficient rhombohedral hafnium oxide, from measurement and from simulation.

The DFT-simulated equivalent *r*-HfO_1.5_ (as already described in Section 3.1) has been relaxed from a cubic structure (α = β = γ = 90°) with a lattice parameter close to the measured value as calculated from the measured (002) reflection. Strikingly, also for the simulated phase, a peak splitting of (111) into (111) and (1̅11) is evident after relaxation (in agreement with the previously discussed experimental results).

The table in Figure 2e gives an overview of the subtle variations of the stoichiometric reference as well as the measured and simulated deficient structure yielding highly similar lattice parameters and unit cell volumes. Further, the differences for the relevant lattice planes (111), (1̅11), and (002) are listed with the last row indicating at which angle Ψ the lattice planes are located relative to the (111) reflection. As the (111) reflection corresponds to the out-of-plane growth direction (see Figure 1a), the Ψ angle is directly translated into the pole-figure measurements as shown in Figure 2f,g. The pole figure shown in Figure 2f—being captured at 31° in 2θ—shows a distinct pseudo-sixfold symmetry at Ψ ∼ 70.7°, which can be assigned to (1̅11) planes. As the rhombohedral system is limited to a threefold symmetry, the six reflections are the result of two domains being offset by 60° in φ and indexed with distinct black and white labeling. Similarly, the pole figure shown in Figure 2g—being captured at 35.5° in 2θ—shows reflections at Ψ ∼ 55.0°, which are accordingly indexed to two domains of (002) planes. Previous TEM investigations showed that these domains are of single-crystalline quality with dimensions of several 10 nm.^20^ The high-resolution phase contrast showed the high crystallinity of all hafnium oxide thin films ruling out amorphous metallic or suboxide clusters.^20^

A visual comparison between the stoichiometric cubic reference and the DFT-simulated phase of *r*-HfO_1.5_ is shown in Figure 2h,i. While Figure 2h shows the *Fm*3*®m* stoichiometric phase with eight oxygen atoms per unit cell, Figure 2i shows the simulated rhombohedral phase with two oxygen vacancies per unit cell (compare Table S1 in the Supporting Information). Figure 2j,k shows the phase along the (111) growth direction from top- and side-view perspective, highlighting the threefold symmetry as previously discussed for the (1̅11) and (002) planes.

To our knowledge, the here described phase is the first reported rhombohedral phase in pure hafnium oxide so far. It is important to mention that a variety of similar rhombohedral structures have recently been observed in epitaxially grown hafnium zirconium oxide (HZO) thin films.^33−35^ These rhombohedral HZO phases showed ferroelectric properties. Due to the high conductivity of our thin films, it is impossible to measure polar *P*–*E* loops (which is a vital criterion to confirm ferroelectric behavior).^36^ However, the calculated space group of **R**3*m* (see Table S1 in the Supporting Information) is of polar symmetry.^37^ Therefore, both results on *r*-HZO and (the here reported) *r*-HfO_2–*x*_ are in good agreement and indicate that this structural transition in hafnium oxide is possible through either substitution (with zirconium) or by inducing oxygen deficiency (through vacancies). Another important distinction is that in the cases of *r*-HZO, the rhombohedral distortion was attributed to strain effects from the substrate.^33−35^ Here, we show via DFT calculations (as previously discussed) that *r*-HfO_1.5_ may exist as a thermodynamically stable compound.

### 3.3 Band Structure and Spectroscopic Results Compared to the Calculated DOS of *m*-HfO_2_ and *r*-HfO_2–*x*_

Section 3.2 shows excellent agreement between measured and simulated structural data for *r*-HfO_2–*x*_. In this section, we discuss the band structure as obtained from spectroscopic results (UV–vis transmission and photoelectron spectroscopy) and theoretical densities of states (DOSs) from DFT simulations. For this purpose, the theoretical and experimental data for the stoichiometric monoclinic as well as the oxygen-deficient rhombohedral case are compared. For the monoclinic stoichiometric case, [O]/[Hf]=2 (sample #1) applies, while for the deficient rhombohedral case, a composition of [O]/[Hf]=1.7 (sample #5) according to XPS estimation and [O]/[Hf]=1.5 for the DFT simulation apply. As mentioned in Section 3.1, the composition of HfO_1.5_ (close to the experimental estimate of HfO_1.7_) was identified as the critical composition for the transition from monoclinic to rhombohedral structure.

**Figure 3:**
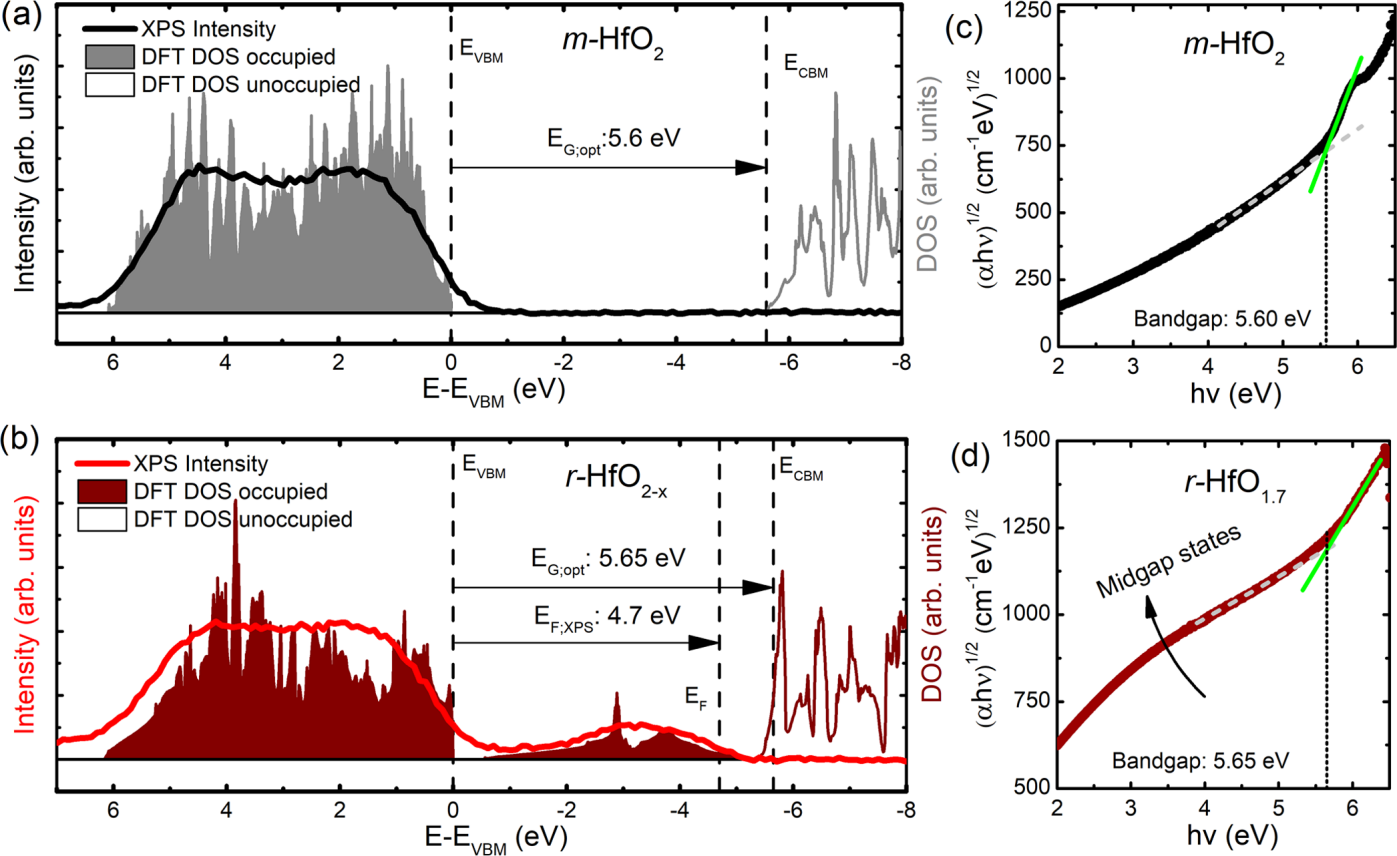
Comparison of DFT-simulated DOS with experimental spectral results from XPS and bandgap from optical transmission spectroscopy for (a) *m*-HfO_2_ and (b) *r*-HfO_1.7_ (measured) and *r*-HfO_1.5_ (calculated). Note the high conformity between measurement and simulation in both cases. Further, the calculated occupied versus unoccupied states are in good agreement with the XPS-estimated Fermi level position. As a result of the oxygen vacancies, a defect band emerges in the previously unoccupied bandgap, providing states in the vicinity of the Fermi level, therefore promoting electrical conduction. (c), (d) Corresponding Tauc plots showing the origin of the bandgaps for both phases with baseline correction.

Figure 3a shows the calculated total DOS displaying occupied and unoccupied states of monoclinic HfO_2_ being separated by an insulating bandgap (as expected for stoichiometric hafnia). By plotting the spectroscopic data together with the calculated total DOS, a high level of agreement becomes apparent. While the XPS data resemble the states of the valence band, it is limited to states with positive binding energy starting from the Fermi level. By estimating the optical bandgap via UV–vis measurements, one can deduce the energy difference from the valence band maximum (VBM) to conduction band minimum (CBM). As the calculated states are aligned to the VBM, the bandgap can be directly read from the abscissa. Here, the optical bandgap (dashed line) with 5.60 eV is in excellent agreement with the appearance of unoccupied DOS as calculated by DFT simulation at 5.56 eV above the Fermi level. This is achieved through the use of the hybrid functional HSE06, which allows a better estimate of bandgaps than the usual GGA functional as discussed in further detail in Section 3.4.

As shown in Figure 3b, the agreement between experimental data and DFT calculations for rhombohedral *r*-HfO_2–*x*_ is very good. Also, in this case, the spectroscopic data resembles the calculated DOS well. In particular, the DFT calculations also show the appearance of dispersive states in the bandgap over several eV. As the DOS in the bandgap is comparably small, the absorption edge for the corresponding optical bandgap can still be clearly identified in the absorption spectra (as will be discussed later). This is reflected in Figure 3b, as the optical bandgap of 5.65 eV is in excellent agreement with the energetic difference between the most prominent occupied vs unoccupied DOS with 5.41 eV.

Further, the location of the experimentally determined Fermi level (*E*_F,XPS_, corresponding to the temperature-dependent chemical potential) is above the vast majority of midgap states. This finding is also in agreement with the obtained DFT results. However, the calculated Fermi level (for *T* = 0 K) is above all midgap states up to the conduction band, the position of the (experimental) chemical potential rather cuts those defect bands at 4.7 eV with respect to the reference point *E*_VBM_. In any case, the Fermi level appears in the vicinity of oxygen vacancy-induced midgap states, therefore promoting significant electrical conduction of *r*-HfO_2–*x*_ as previously discovered by resistance and Hall effect measurements.^20^

Figure 3c,d shows the Tauc plots of the corresponding samples for *m*-HfO_2_ (sample #1) and *r*-HfO_1.7_ (sample #5) as obtained from UV–vis transmission measurements. The analysis in the Tauc approach gives the following relation between absorption α, energy *h*ν, bandgap *E*_g_, and corresponding slope *A*
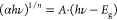
where *n* is chosen according to the type of bandgap with *n* = 1/2 for allowed direct and *n* = 2 for allowed indirect transitions.^38,39^ In accordance with a previous publication, *n* = 2 is chosen for the ordinate^20^ in the presented graphs as *m*-HfO_2_ was shown to have an indirect bandgap by several calculations.^40−42^ For both, stoichiometric *m*-HfO_2_ and *c*-HfO_2_ (close to the *r*-HfO_2–*x*_ structure), the direct and indirect transitions are found to be almost degenerate in energy.^40−42^ In Section 3.4, we confirm the energetic degeneracy with precise hybrid functional calculations where we find direct and indirect transitions for *m*-HfO_2_ and *r*-HfO_1.5_, which are extremely close in energy. For both *m*-HfO_2_ and *r*-HfO_1.7_, a clear linear absorption slope can be discerned. While *r*-HfO_1.7_ shows only one slope, *m*-HfO_2_ shows two absorption features, which are well known to be inherent to the monoclinic phase.^20,43−45^ Further, in both cases, a baseline absorption becomes apparent. For *r*-HfO_1.7_, the increased baseline absorption is significantly stronger than for *m*-HfO_2_, which is a consequence of the midgap states (as measured and calculated), which extend over the gap between the most prominent DOS. For *m*-HfO_2_ on the other hand (as measured and calculated), no states are visible in the bandgap. Therefore, the baseline absorption is most likely a consequence of a modified reflection or scattering of the Sapphire substrate after the hafnia thin film deposition.^46^ In any case, an appropriate baseline correction needs to be applied to account for the above-mentioned physical effects. Extrapolating the absorption slope to the extrapolated baseline absorption instead of the abscissa is a proven and reliable method for optical bandgap estimation.^47^ By applying this method, the extracted values of 5.6 and 5.65 eV for *m*-HfO_2_ and *r*-HfO_1.7_, respectively, are—as previously mentioned—in excellent agreement with the discussed band structure calculations of 5.56 eV for *m*-HfO_2_ and 5.41 eV for *r*-HfO_1.5_.

### 3.4 Analysis of the Electronic Structures of Oxygen-Deficient HfO_2–*x*_

**Figure 4:**
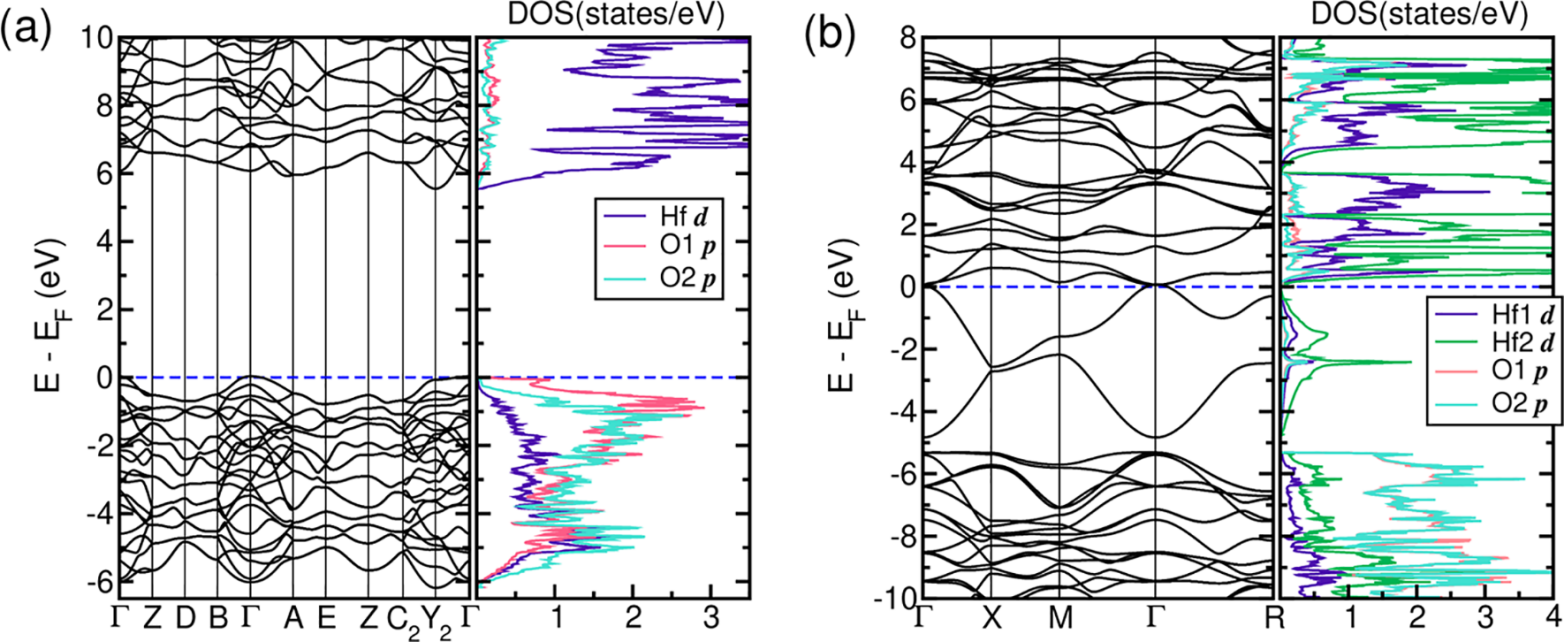
Band structures and orbital-resolved DOSs for (a) *m*-HfO_2_ and (b) *r*-HfO_1.5_ within HSE06. The calculated bandgap in *m*-HfO_2_ is 5.56 eV, which is in good agreement with our measured bandgap of 5.6 eV. In *r*-HfO_1.5_, two dispersive bands appear below the Fermi level resulting from oxygen vacancies. These defect states consist of mixed orbitals of Hf 5d and O 2p.

In this section, we provide a detailed description of the calculated electronic structures in HfO_2–*x*_. While regular exchange–correlation functionals such as the local density approximation and the generalized gradient approximation, which are widely used in DFT calculations, substantially underestimated the bandgap of monoclinic HfO_2_,^40,41,48^ it was already shown that the use of the GW method as well as hybrid functionals like B3LYP and PBE0 can provide satisfying results.^42,49,50^ As demonstrated in the previous section, we show that the observed bandgap can be precisely estimated by adopting the hybrid functional method HSE06 as implemented in VASP. Figure 4a shows calculated band structures and orbital-resolved DOSs in monoclinic HfO_2_ within HSE06. Fully occupied oxygen bands show significant hybridized characters with Hf 5d states. On the other hand, empty Hf 5d states arise above 5.6 eV. Similar electronic structures occur in stoichiometric *c*-HfO_2_ with a bandgap of 5.46 eV, as shown in Figure S2b. In this case, unoccupied Hf 5d states are split into lower e_g_ and higher t_2g_ states due to the crystal field environment of Hf in a cube of neighboring O. Furthermore, the band width of fully occupied oxygen 2*p* states in *c*-HfO_2_ is slightly larger by about 1 eV than that in *m*-HfO_2_. The VBM in *m*-HfO_2_ happens at the Γ point as well as the Y_2_(−π/*a*, 0,0) point, which is nearly degenerate in energy with the Γ point. On the other hand, the CBM appears at the Y_2_ point, which gives rise to a possible combination of direct and indirect bandgaps. In agreement with our experimental results, we find that *m-*HfO_2_ is more energetically stable than *c*-HfO_2_ by 181.5 meV within GGA and 223 meV within HSE06 (see Figure 1d).

Figure 4b illustrates a band structure and orbital-resolved DOSs in *r*-HfO_1.5_, which is in good agreement with our experimental results, within HSE06 as discussed in Section 3.3. For comparison, we show the electronic structure of stoichiometric HfO_2_ in Figure 4a. Two dispersive bands arise from the oxygen deficiency (HfO_2–*x*_, *x* = 0.5; Hf 5d^+δ^, δ = 1) appearing from 0 to −5 eV below the Fermi level. Note that each band includes two spins, and there are four Hf atoms in the unit cell for the calculations. Those midgap bands consist of hybridized states between Hf 5d and O 2p. Interestingly, the top of these midgap states is touching the conduction Hf 5d states at the Γ point, making this system more metallic. On the other hand, there is still a small gap between these midgap states and fully occupied O 2p bands with a direct gap at the Γ point by about 482 meV, which hinders this system to be entirely metallic. Regarding bandgaps, both direct and indirect transitions appear to be possible from either the Γ or *X* point of the occupied O 2p bands maximum and the Γ point of the midgap states minimum, respectively, as shown in Figure 4b. Contrary to *r*-HfO_1.5_, the midgap states in *m*-HfO_1.5_ within HSE06 (see Figure S2a) lie between the occupied O 2p and unoccupied Hf 5d states. These results are consistent with the previously measured resistivity on *r*-HfO_1.7_ (there denoted LTP c-HfO_1.7_), which is more conductive than *m*-HfO_2–x_.^20^ Further, the difference in total energies within HSE06 between *r*-HfO_1.5_ and *m*-HfO_1.5_ is almost eliminated, as shown in Figure 1d. Note that the total energy within HSE06 of *m*-HfO_1.5_ is lower than *r*-HfO_1.5_ by 1.6 meV, but the value is marginal and compatible with thermal effects. (In the GGA results, *r*-HfO_1.5_ is even energetically favored over *m*-HfO_1.5_ by about 18 meV.) Therefore, our DFT results clearly confirm our experimental observations that oxygen vacancies stabilize the rhombohedral structure of hafnium oxide and lead to the significant conductivity of this phase.

## 4 Conclusions

This work presents detailed experimental and theoretical crystal and electronic structure investigations for a recently discovered intermediate substoichiometric phase between hafnia and hafnium. We show that this phase is stabilized by oxygen vacancies beyond a critical concentration and is of rhombohedral nature (*r*-HfO_2–*x*_). Through total energy calculations within DFT (HSE06), we find that the introduction of oxygen vacancies in the monoclinic phase leads to a preferential formation of the rhombohedral phase. The calculated rhombohedral structure of pure hafnium oxide belongs to the polar space group **R**3**m**, showing similarities to a recently discovered rhombohedral ferroelectric hafnium zircon oxide (HZO) phase. The DFT calculations show that *r*-HfO_2–*x*_ is not epitaxially induced but a stable oxygen-deficient phase. Additionally, by comparing the bandgap and spectral data of the valence band region with the density of states calculated via HSE06 for *r*-HfO_2–*x*_ we find excellent agreement. We have shown that oxygen vacancy-induced midgap states are present for the rhombohedral phase, being the origin of the recently discovered electrical conductance and midgap optical absorption for this phase. Finally, the calculated orbital-resolved DOSs highlight the oxygen vacancy-dependent hybridization of hafnium and oxygen orbitals.

## Supporting Information Available

Oxygen plasma intensity and chamber pressure during deposition (S1); electronic structures of *m*- and *c*-HfO_1.75_ (S2); XPS spectra used for stoichiometry estimation (S3); and potential influences on the deviation of a single (1̅11) reflection (S4) (PDF)
