# Supplementary material for: Crystal and Electronic Structure of Oxygen Vacancy Stabilized Rhombohedral Hafnium Oxide
Source: ACS Appl Electron Mater. 2023 Jan 26;5(2):754–63. doi: 10.1021/acsaelm.2c01255 (PMC9979600; doi:10.1021/acsaelm.2c01255)
Supplement: Supplementary file 1 — el2c01255_si_001.pdf [file el2c01255_si_001.pdf]

Supporting Information

Crystal and electronic structure of oxygen vacancy stabilized rhombohedral hafnium oxide

Nico Kaiser[1]‡, Young-Joon Song[2]‡, Tobias Vogel[1], Eszter Piros[1], Taewook Kim[1], Philipp Schreyer[1], Stefan Petzold[1], Roser Valentí[2] and Lambert Alff[1]*

* corresponding author (nico.kaiser@tu-darmstadt.de); ‡ these authors contributed equally

1 Advanced Thin Film Technology Division, Institute of Materials Science, TU Darmstadt, Alarich-Weiss-Str. 2, 64287 Darmstadt, Germany;

2 Institute for Theoretical Physics, Goethe-University Frankfurt, Max-von-Laue-Straße 1, 60438 Frankfurt am Main, Germany

S1. Oxygen plasma intensity and chamber pressure during deposition

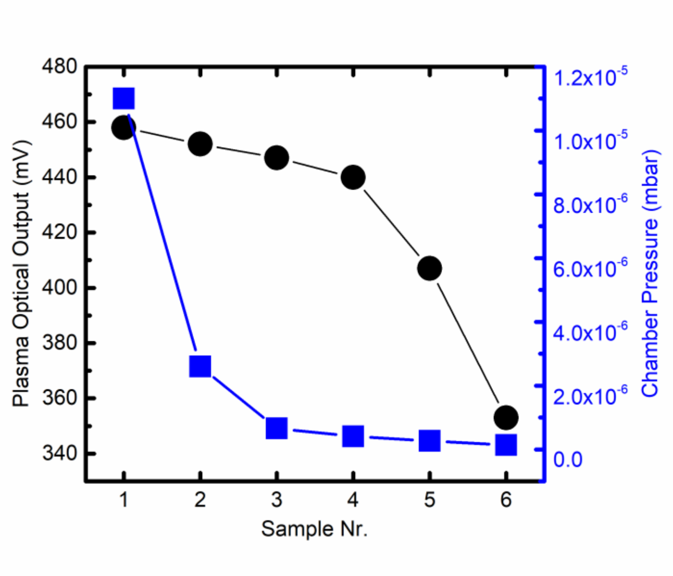

Figure S1. Plasma intensity as indicated by the optical output potential and the chamber pressure for all investigated samples show non-linearity to the linear deposition conditions of hafnium evaporation rate and oxygen gas flow. Especially the rather strong decrease of oxygen plasma intensity from sample #4 over sample #5 to #6 may be responsible for the similarly strong change in the samples oxygen content.

S2. Electronic structures of *m*- and *c*-$HfO_{1.75}$

As mentioned in the main text, oxygen vacancies give rise to midgap states between the VBM and the CBM. A single midgap band arises in both *m*- and *r*-$HfO_{1.75}$ just below the Fermi level, as shown in figure S1. The difference in the band width of the midgap band between *m*- and *r*-$HfO_{1.75}$ is similar to the case between *m*- and *r*-$HfO_{1.5}$. Each midgap band consists of hybridized states between Hf 5*d* and O 2*p*. The total energy in *m*-$HfO_{1.75}$ is still lower than that in *r*-$HfO_{1.75}$ by 83.3 meV in GGA and 102.9 meV in HSE06. (See Figure 1d in the main text). As more oxygen vacancies are present, the total energy difference between *m*- and *r*-HfO_{2-x} gets smaller and smaller. (See the main text.)

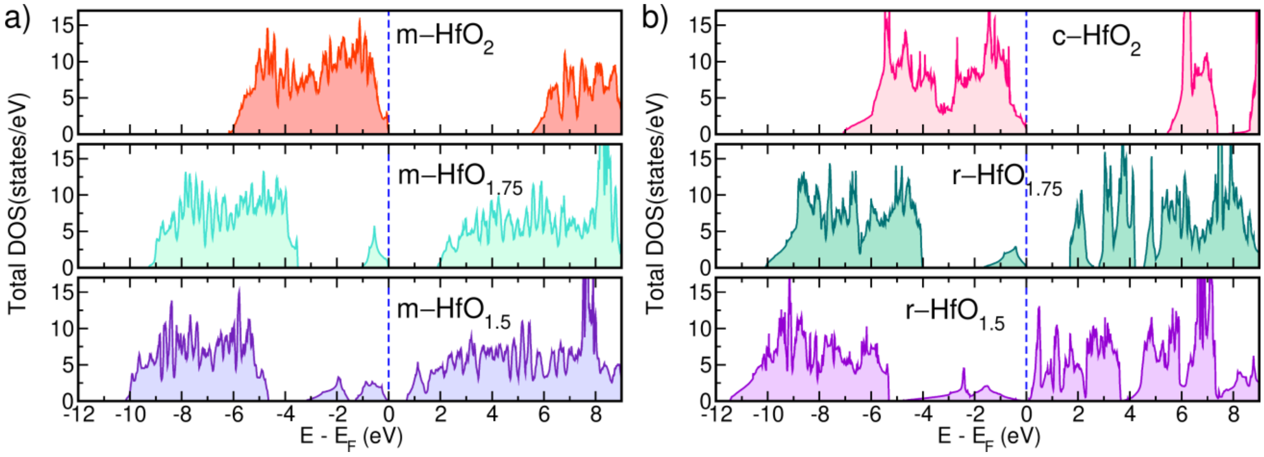

Figure S2. Total density of states in (a) monoclinic and (b) cubic/rhombohedral HfO_{2-x} within HSE06. In both structures, midgap states arises below the Fermi level, as a consequence of the presence of oxygen vacancies. Interestingly, the midgap states in *r*-HfO_{2-x} show a larger band width than that in *m*-HfO_{2-x}.

$m\!-\!\mathbf{HfO}_2$ ($P2_1$/c)

a	b	c	α	β	γ
5.07849	5.16438	5.23505	90	99.6675	90

Atom	Positions	Site
Hf	(0.22231, 0.04278, 0.79287)	(4e)
O1	(0.42691, 0.34104, 0.66244)	(4e)
O2	(0.05327, 0.75862, 0.51760)	(4e)

$c\!-\!\mathbf{HfO}_2$ (Fm-$3m$)

a
5.01691

Atom	Positions	Site
Hf	(0.00000, 0.00000, 0.00000)	(4a)
O	(0.75000, 0.25000, 0.25000)	(8c)

$m\!-\!\mathbf{HfO}_{1.75}$ ($P1$)

a	b	c	α	β	γ
5.03468	5.11268	5.19333	90.2094	90.0281	98.8509

Atom	Positions	Site
Hf1	(0.99340, 0.50010, 0.41363)	(1a)
Hf2	(0.44093, 0.08184, 0.50281)	(1a)
Hf3	(0.44261, 0.58047, 0.91722)	(1a)
Hf4	(0.00000, 0.00000, 0.00000)	(1a)
O1	(0.80645, 0.60789, 0.09245)	(1a)
O2	(0.64076, 0.97266, 0.81588)	(1a)
O3	(0.63208, 0.47155, 0.59542)	(1a)
O4	(0.80632, 0.11288, 0.32068)	(1a)
O5	(0.16405, 0.77434, 0.69848)	(1a)
O6	(0.27468, 0.80463, 0.21886)	(1a)
O7	(0.27202, 0.30409, 0.19115)	(1a)

$r\!-\!\mathbf{HfO}_{1.75}$ ($R3m$)

a	α
5.02028	90.5356

Atom	Positions	Site
Hf1	(0.49017, 0.49017, 0.49017)	(1a)
Hf2	(0.00000, 0.00000, 0.50716)	(3b)
O1	(0.78418, 0.78418, 0.78418)	(1a)
O2	(0.27350, 0.72777, 0.72777)	(3b)
O3	(0.22536, 0.78739, 0.22536)	(3b)

$m\!-\!\mathbf{HfO}_{1.5}$ ($P1$)

a	b	c	α	β	γ
4.99658	5.10462	5.15127	89.4642	89.3485	81.4499

Atom	Positions	Site
Hf1	(0.98651, 0.50475, 0.58696)	(1a)
Hf2	(0.43559, 0.92500, 0.49065)	(1a)
Hf3	(0.43192, 0.43086, 0.07895)	(1a)
Hf4	(0.00000, 0.00000, 0.00000)	(1a)
O1	(0.64059, 0.04991, 0.18027)	(1a)
O2	(0.63561, 0.53833, 0.38867)	(1a)
O3	(0.80263, 0.88392, 0.68165)	(1a)
O4	(0.15896, 0.22810, 0.29851)	(1a)
O5	(0.26490, 0.20362, 0.77751)	(1a)
O6	(0.27749, 0.69810, 0.79573)	(1a)

$r\!-\!\mathbf{HfO}_{1.5}$ ($R3m$)

a	α
4.96722	89.3739

Atom	Positions	Site
Hf1	(0.98226, 0.98226, 0.98226)	(1a)
Hf2	(0.50232, 0.50232, 0.01942)	(3b)
O1	(0.23521, 0.23521, 0.77455)	(3b)
O2	(0.73280, 0.73280, 0.27712)	(3b)

Table S1: Fully relaxed structural data within DFT. Given are lattice parameters (Å), angles (°), and atomic positions (fractional coordinates) in monoclinic and cubic/rhombohedral HfO_{2-x} (x = 0, 0.25, and 0.5) within GGA. Oxygen deficiency in cubic HfO_{2-x} leads to a tiny rhombohedral

distortion. On the other hand, monoclinic HfO_{2-x} reduces to triclinic symmetry under oxygen deficiency.

S3. XPS spectra used for stoichiometry estimation

Figure S3 shows the XPS spectra of the investigated samples. A Shirley-type background was subtracted from the spectra. Hf^{4+} and Hf^{x+} contributions have been fitted via Gaussian-Lorentzian functions while Hf^0 signals have been fitted by using Doniac-Sunjic functions. Note that the Hf *4f* emission line of monoclinic sample #1 could be fitted with a single doublet, representing Hf^{4+} states only (as expected for a highly stoichiometric sample), while or more deficient samples show additional Hf^{x+} suboxide-signals. For the most deficient sample a clear contribution of Hf^0 states becomes prominent. The spectra of the O *1s* orbitals can be fitted by a main contribution, which can be assigned to lattice oxygen, while the small shoulder is commonly attributed to surface oxygen e.g. due to residual water adsorbates in the system. The surface contribution was accordingly subtracted for the stoichiometry estimation.

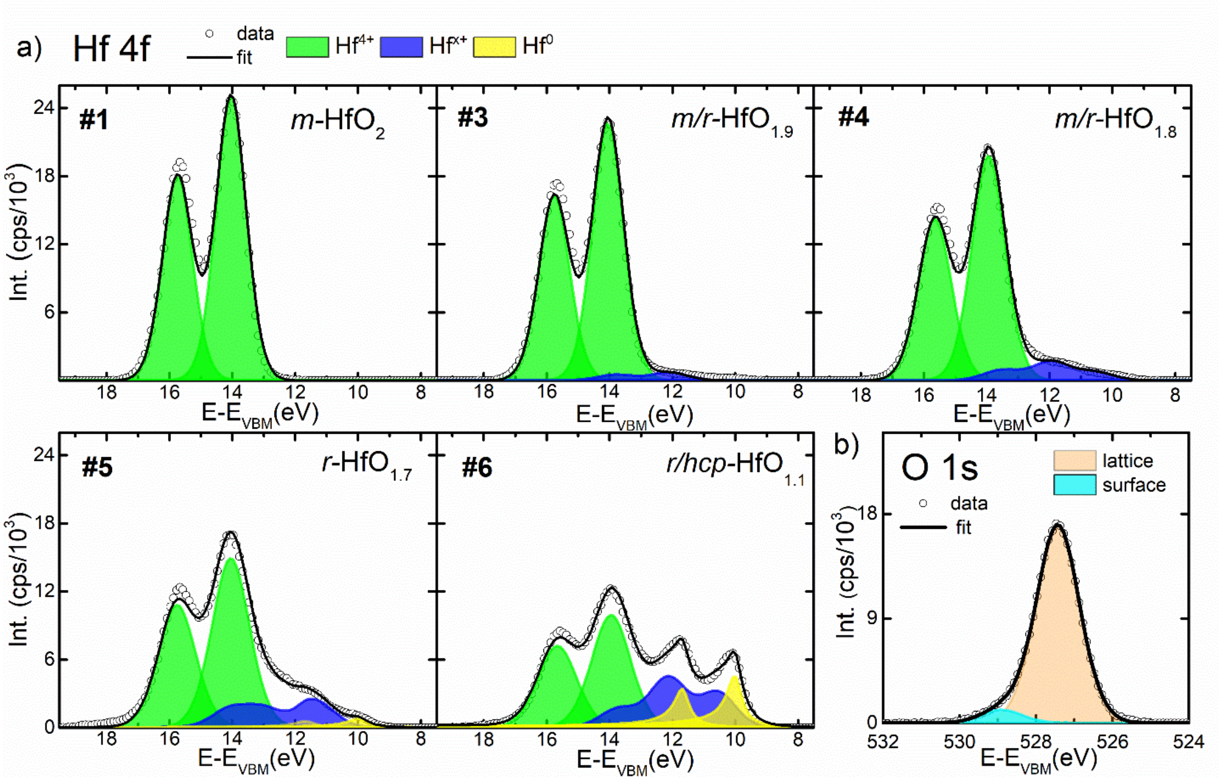

Figure S3. XPS spectra of a) Hf *4f* emission lines of samples #1 and #3 - #6 as well as b) a representative O *1s* emission line from sample *r*-$HfO_{1.7}$ #5.

S4. Potential influences on the deviation of a single (-111) reflection

In the main text, it was discussed that one single (-111) reflection showed a deviation of ~ 0.2° in 2θ. However, this is not possible due to crystallographic symmetry. Here we discuss potential influences for this deviation with the help of additional measurements. Figure S2 shows that the deviation is neither the result of misalignment, nor does it come from the XRD setup. Also, generally no λ/n contamination is expected as all measurements have been performed with a D/teX 250 unit from Rigaku which has an appropriate limited energy window to filter such artifacts. An influence from the substrate can also be excluded since no relevant reflexes are expected close to 30° in 2θ. Therefore, the most likely explanation for this shift is an additional unidentified structural (separate) contribution from the thin film or its interface to the substrate.

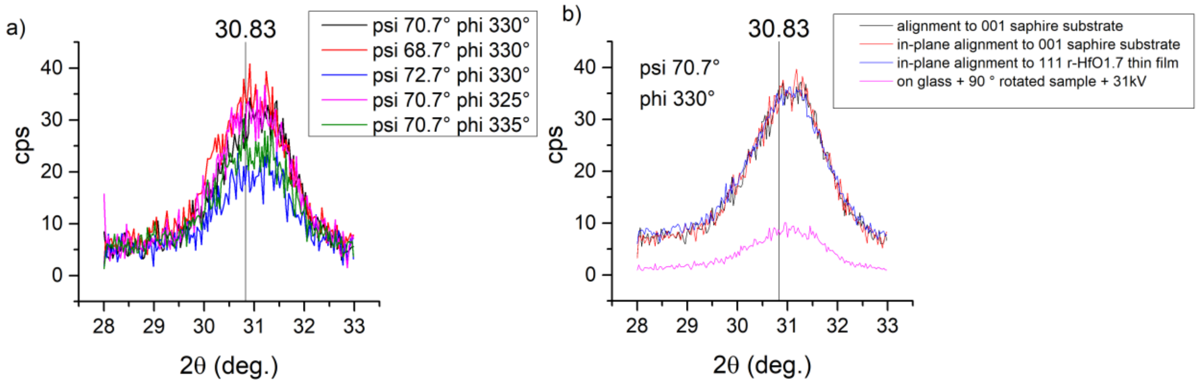

Figure S4: a) Shows that the deviation of ~ 0.2° in 2θ for one (-111) reflection (expected at 30.83 ° in 2θ) persists over considerable area in psi and phi and b) shows that independent of the applied alignment, no change of the deviation can be observed. b) also includes one measurement in pink, which shows that the deviation even persists with a glass plate as support, and also if the sample is rotated by 90° the reflex position does not change (same relative phi position with respect to the sample). Therefore, the deviation is not the consequence of an alignment error and does not originate from the goniometer setup. The reduced intensity for the pink scan can be attributed to a reduced acceleration voltage of 31 kV to exclude λ/4 contamination (which is therefore shown to have no influence).
